# Exercise-Induced Pulmonary Edema in a Triathlon

**DOI:** 10.1155/2015/968152

**Published:** 2015-07-02

**Authors:** Hirotomo Yamanashi, Jun Koyamatsu, Masaharu Nobuyoshi, Kunihiko Murase, Takahiro Maeda

**Affiliations:** ^1^Department of Island and Community Medicine, Nagasaki University Graduate School of Biomedical Science, Goto, Nagasaki 853-8691, Japan; ^2^Goto Central Hospital, Yoshikugi, Goto, Nagasaki 853-8691, Japan

## Abstract

*Introduction*. Family physicians have more opportunities to attend athletic competitions as medical staff at first-aid centers because of the increasing popularity of endurance sports. *Case*. A 38-year-old man who participated in a triathlon race experienced difficulty in breathing after swimming and was moved to a first-aid center. His initial oxygen saturation was 82% and a thoracic computed tomography scan showed bilateral ground glass opacity in the peripheral lungs. His diagnosis was noncardiogenic pulmonary edema associated with exercise or swimming: exercise-induced pulmonary edema (EIPE) or swimming-induced pulmonary edema (SIPE). Treatment with furosemide and corticosteroid relieved his symptoms of pulmonary edema. *Discussion*. Noncardiogenic pulmonary edema associated with endurance sports is not common, but knowledge about EIPE/SIPE or neurogenic pulmonary edema associated with hyponatremia, which is called Ayus-Arieff syndrome, is crucial. Knowledge and caution for possible risk factors, such as exposure to cold water or overhydration, are essential for both medical staff and endurance athletes. *Conclusion*. To determine the presence of pulmonary edema associated with strenuous exercise, oxygen saturation should be used as a screening tool at a first-aid center. To avoid risks for EIPE/SIPE, knowledge about these diseases is essential for medical staff and for athletes who perform extreme exercise.

## 1. Introduction

Family physicians frequently attend athletic competitions as medical assistants at first-aid centers because of the increasing popularity of endurance sports. Noncardiogenic pulmonary edema associated with exercise has been previously reported in various endurance athletes, including swimmers [1–4], divers [5, 6], marathon runners [7], triathletes [8], and cyclists [9]. The mechanism of this condition is unclear [10], but cases are categorized into exercise-induced pulmonary edema (EIPE), swimming-induced pulmonary edema (SIPE), or neurogenic pulmonary edema associated with hyponatremia, which is also known as Ayus-Arieff syndrome [7, 11, 12]. The prognosis of EIPE/SIPE is generally good with a quick diagnosis and adequate treatment, whereas an unfavorable prognosis has been reported with neurogenic pulmonary edema [13].

## 2. Case Presentation

A 38-year-old man participated in a triathlon race, which consisted of 3.8 km of swimming, 180 km of cycling, and 42.2 km of running. Ambient and water temperatures were 20.0°C and 21.4°C, respectively. Because he appeared to have difficulty in breathing during swimming, lifeguards moved him to the first-aid center shortly after he reached the coast. He had no relevant past medical or allergic history. He denied submersion injury or overt aspiration of seawater.

On physical examination, his consciousness was alert, blood pressure was 119/84 mm Hg, respiratory rate was 14 breaths/min, and initial oxygen saturation was 82% at room air. A respiratory examination showed bilateral wheezing with forced breathing. A standby ambulance quickly delivered him to the nearby hospital. Remarkable findings included bilateral ground glass opacity in the peripheral lungs on a thoracic computed tomography scan. A laboratory examination showed a serum sodium level of 143.9 mEq/L and B-type natriuretic peptide level of 11.5 pg/mL.

We diagnosed this case as EIPE/SIPE because there was no elevation in cardiac enzymes, with a normal electrocardiogram after consideration of the possibility of neurogenic pulmonary edema associated with hyponatremia or cardiogenic pulmonary edema. We treated him with methylprednisolone and furosemide intravenously. All symptoms of pulmonary edema, including dyspnea and hypoxemia, resolved within 4.5 hours, and he was discharged the same day without recurrence.

We have experienced six EIPE/SIPE cases at a triathlon race over a decade (Table 1). Among six men (age: 28–57 years), all completely recovered shortly after admission (admission period: 1–10 days), except for one patient who needed mechanical ventilation. Chest X-rays showed diffuse consolidation in all of the patients. Hyponatremia was not observed in any patients.

## 3. Discussion

The mechanism of EIPE/SIPE is not well known, but stress failure of pulmonary capillaries likely plays a major role in development of pulmonary edema in scuba divers and swimmers [1–6]. Constriction of peripheral blood vessels caused by exposure to a cold environment to maintain a core temperature leads to central pooling of blood. This leads to increased preload, pulmonary artery pressure, and cardiac output, resulting in a rise in pulmonary capillary pressure. Excessive drinking of water to counteract dehydration also causes an increase in preload and pulmonary capillary pressure. These physiological responses appear to cause failure of pulmonary capillaries and contribute to development of acute pulmonary edema.

Knowledge on EIPE/SIPE is essential for relevant medical staff and endurance athletes. A pulse oximeter should be actively used as an available screening tool to detect EIPE/SIPE at the first-aid center. However, the initial presentation of EIPE/SIPE is difficult to distinguish from cardiogenic pulmonary edema or Ayus-Arieff syndrome without biochemical investigations at a first-aid center. Therefore, medical personnel should quickly detect hypoxic patients, and further assessment should be performed at a hospital. To avoid risks for EIPE/SIPE, knowledge on factors contributing to development of pulmonary edema, such as exposure to a cold environment and excessive drinking of water, is essential [14, 15]. Careful instructions should be provided to participants in endurance sports because one study reported recurrent SIPE episodes as high as 22.9% [2].

## 4. Conclusion

To rapidly manage patients with potential critical pulmonary edema associated with strenuous exercise, initial assessment of hypoxia is important in the setting of a first-aid center. To avoid risks for EIPE/SIPE, knowledge of these diseases is essential for relevant medical staff and for athletes who perform extreme exercise.

## Figures and Tables

**Table 1:** Characteristics of exercise-induced pulmonary edema or swimming-induced pulmonary edema in triathlons.

Case number	Age (y)	Sex	Chief complaint	Past history	Onset	Oxygen saturation (%)	Body temperature (°C)	Sodium level (mEq/L)	Ejection fraction by ultrasonography (%)	Admission period (d)
1	38	Male	Dyspnea	None	After finishing swimming	82%	36.8	143.9	Not examined	1

2	57	Male	Dyspnea, chill	None	After swimming for 30 minutes	75%	36.3	143.0	73	4

3	28	Male	Dyspnea, nausea, and fatigue	None	Abstention in the marathon	54%	NA	141.6	67.5	10

4	39	Male	Dyspnea, hemosputum	Cough for 1 week	Abstention in the competition	87%	38.7	142.5	72	3

5	49	Male	Dyspnea	None	Hypoxia recognized at the end of competition	77%	37.3	142.9	68	2

6	40	Male	Dyspnea, cough	None	Hypoxia recognized at the end of competition	85%	36.8	134.8	54	3

NA indicates not available.
